# Retrospective analysis of autoimmune bullous diseases in Middle Franconia

**DOI:** 10.3389/fimmu.2023.1256617

**Published:** 2023-10-10

**Authors:** Lukas Sollfrank, Valerie Schönfelder, Micheal Sticherling

**Affiliations:** ^1^ Department of Dermatology, University Hospital Erlangen, Erlangen, Germany; ^2^ Deutsches Zentrum Immuntherapie, Medizinische Fakultät, Friedrich-Alexander-Universität Erlangen-Nürnberg, Erlangen, Germany

**Keywords:** autoimmune bullous dermatoses, bullous dermatoses, pemphigus diseases, pemphigoid diseases, epidemiology, bullous pemphigod

## Abstract

**Introduction:**

Autoimmune bullous diseases (AIBDs) are a group of rare cutaneous disorders affecting cornified skin and mucous membranes. They are characterized by tense or flaccid blistering and erosions due to autoantibodies against desmosomal and hemidesmosomal structural proteins of the skin. This group of disorders can be divided into those of pemphigoid and those of pemphigus diseases. If left untreated, these autoimmune diseases can cause serious or even life-threatening complications such as loss of fluid, superinfections or impaired food intake. Due to modern standardized serological assays, the diagnosis of AIBDs can usually be confirmed in combination with their clinical appearance. Whereas for a long time corticosteroids were the major players in the treatment of these diseases, with the approval of rituximab and other immunosuppressive agents, the therapy has increasingly improved.

**Methods:**

In this study, we aimed to investigate epidemiologic and clinical features as well as diagnostics and therapy of bullous autoimmune diseases in Middle Franconia, a governorate within the German federal state of Bavaria. Patients diagnosed or treated because of a AIBDs between 01.04.2013 and 31.03.2019 at the dermatological department of the university hospital Erlangen were included in this retrospective study (n = 242). Patients were either diagnosed for the first time (n=176) or the diagnosis has been confirmed (n=66) at the department. The respective incidence was calculated among the 176 subjects who had been diagnosed at the center in this period. Data was taken from patient records and analyzed with Microsoft® Excel. The evaluation included the diagnoses of pemphigus vulgaris (PV), pemphigus foliaceus (PF), bullous pemphigoid (BP), mucous membrane pemphigoid (MMP), linear IgA dermatosis (LAD), epidermolysis bullosa acquisita (EBA), and dermatitis herpetiformis (DH).

**Results:**

This study shows that the incidence of each AIBDs in Middle Franconia is low and comparable (PV, PF, LAD, EBA) or lower (BP, MMP, DH) than in other studies and regions. BP is the most common newly diagnosed AIBD in Middle Franconia.

**Discussion:**

Due to the chronic and sometimes severe course of AIBDs, repeated in-house treatments are often necessary. To date, mainly topically and systemically applied corticosteroids in combination with immunomodulators are used as first-line therapy.

## 1 Introduction

Autoimmune bullous diseases (AIBDs) are rare, autoimmune-induced, organ-specific diseases of the skin and skin-related mucous membranes induced by circulating autoantibodies against structural protein. Depending on the type of disease and its severity, the clinical features range from erythema on the skin and mucous membranes to excoriations, vesicles, blisters or even erosions. In patients with severe disease progression, old age or inadequate therapy, these diseases can lead to increased mortality (1, 2). A rough classification is made into the group of pemphigoid diseases showing erythema, eczema, tense blisters and erosions of the skin and pemphigus diseases presenting more flaccid blistering and erosions. This differentiation was first described clinically and histopathologically by Lever in 1953 (3). In pemphigoid disorders, autoantibodies are directed against proteins of the basement membrane connecting epidermis and dermis whilst in pemphigus diseases, antibodies are directed against desmosomal proteins connecting adjacent epithelial cells (4, 5). DH is a cutaneous manifestation of celiac disease induced by gluten and autoantibody response against the tissue transglutaminases 2 and 3. Clinical signs are polymorphic presenting vesicles, papules and macules (6). All of these diseases present diagnostic challenges due to their varied clinical presentations and overlapping histopathological features, and require a multifaceted approach to accurate assessment (7). Due to the often non-specific symptoms such as erythema, burning or itching at the beginning of the diseases, clinical suspicion of AIBD is frequently not raised and the use of specific immunoassays delayed. This leads to a significant delay in diagnosis, especially in AIBDs affecting the mucosa (8). As AIBDs have a significant impact on the quality of life of patients, there is a need for tools to assess the psychosocial health of those affected, underlining the need for comprehensive care beyond clinical assessment (9). The aim of this study is the comprehensive analysis of AIBDs at the Department of Dermatology of the University Hospital Erlangen with the catchment area of Middle Franconia, a governorate within the south-east German federal state of Bavaria. The epidemiology, clinical presentation, diagnostics and therapy of these diseases were examined.

### 1.1 Pemphigoid diseases

Pemphigoid diseases are characterized by the formation of subepidermal blisters along the dermo-epidermal junction zone. Autoantibodies are directed against hemidesmosomal structural proteins, which disrupts the connection of the basal keratinocytes with the basement membrane zone (5). BP is the most common AIBD in Germany with 6.6 to 13.4 newly diagnosed cases/million/year and rises with age of patients (10–12). Main clinical signs are tense blisters and erosions and in 10-20% mucosal involvement (13). In individuals older than 90 years the incidence was found at 87 new cases per 1 million residents for women and 398 new cases per 1 million residents for men. German Men as a group are more often affected than women by 1.9 times (14). In the UK, the incidence of BP was shown to be higher at 42.8 new cases/million/year (15). The other pemphigoid diseases occur less frequently. In Germany MMP has an incidence of 1.1 to 2.0 per million per year and occurs more frequently in women. As the name suggests, it predominantly affects mucous membranes, especially oral and conjunctival mucosa, eventually leading to blindness (16, 17). Approximately 30-40% of patients present symptoms on the skin with erythema, blisters or erosions (18). The mortality risk in BP is increased by a factor of 2.5, in patients with MMP by a factor of 1.7 (19). LAD is even less common with an incidence of 0.7 to 1.0 new cases/million/year (11, 12, 20). Its clinical appearance features tense, clear or hemorrhagic vesicles and blisters in the area of the skin and mucosa, typically in a linear, annular, “crowns of jewels” or rosette-like pattern (21). While BP and MMP mainly affect older patients, LAD is common in children and between the 6th-7th decades of life (22). With an incidence of 0.5 per million, EBA represents the rarest among the supepidermal blistering diseases of the pemphigoid group and has no sex-predilection (10, 11). It is characterized by skin fragility, leading to the presence of tense bullae and erosions. Individuals can be affected regardless of their age (23). The diagnosis of pemphigoid disorders is based on a combination of clinical criteria, serological and direct immunofluorescence microscopy results (5). The gold standard remains the detection of C3 and autoantibody deposition by direct immunoflourescence of a perilesional biopsy. Pemphigoid diseases present with linear deposits in the area of the dermal-epidermal junction. Furthermore, on monkey oesophagus and human saline split skin, linear deposits can also be shown by indirect immunofluorescence (7).

### 1.2 Pemphigus diseases

Pemphigus diseases, in contrast to pemphigoid diseases, are distinguished by intraepidermal and therefore more unstable blister formation. The two most frequent representatives of this group are pemphigus vulgaris (PV) and pemphigus foliaceus (PF) (4). The incidence of PV in Germany ranges from 0.5 to 1.5 per 1 million inhabitants and year (11, 12). Again, a higher incidence in the UK is reported with 6.8 per million and year (15). In contrast to the pemphigoid disorders, the oral mucosa is affected as first manifestation in 75% of patients with PV. Erosions and enanthema are most common (24, 25). In 77,6%, skin involvement follows 4-5 months after the onset of disease, whereas in some rare cases, blistering of the skin is the only manifestation (26, 27). Due to frequent oral mucosal involvement, food intake is severely impaired in this disease group (4). At an incidence rate of 0.5 per million per year, PF is similarly rare in Germany (12). Since in patients with PF only autoantibodies against desmoglein (Dsg) 1, which is not present in mucous membranes, are produced, its only manifestation is on the skin. The intraepidermal blisters are due to the even thinner blister cover compared to PV more flaccid and fragile, sometimes leading to a total absence of blisters, but presence of erosions (28). With 5% of cases, paraneoplastic Pemphigus accounts for only a small proportion of pemphigus and presents a wide range of clinical manifestations because of its varying autoantibodies (**Table 1**) (29). IgA pemphigus, drug-induced pemphigus and the clinical variants of PV, pemphigus vegetans or pemphigus herpetiformis, are rarely diagnosed (30). The risk of mortality is increased by almost four times in patients with PV. In the paraneoplastic variant, the mortality risk is even increased by a factor of 8 (19). The mean age of onset of pemphigus diseases is around 60 years of age. While slightly more women develop PV, the opposite is true for PF (15, 31). As in pemphigoid diseases, the diagnosis is based on the clinical picture, as well as serologic and direct immunofluorescence microscopy results (4). In pemphigoid diseases, the deposition of C3 and autoantibodies in intraepidermal-epithelial can be observed. In indirect immunofluorescence, intracellular fluorescence can be detected (7).

**Table 1:** Bullous autoimmune dermatosis and their main target antigens.

Diseases with intraepidermal loss of adhesion Pemphigus diseases	Main target antigens
Pemphigus vulgaris	desmoglein 1, desmoglein 3 (4)
Pemphigus foliaceus	desmoglein 1 (4)
Paraneoplastic pemphigus	envoplakin, periplakin, desmoplakins I and II, plectin, BP230, desmoglein 1, desmoglein 3 (29)
Diseases with subepidermal loss of adhesion Pemphigoid diseases	Main target antigens
Bullous pemphigoid in the strict sense	BP180, BP230 (5)
Other pemphigoid diseases	p200, α6β4-Integrin (5)
Mucous membrane pemphigoid	BP180, laminin-5/laminin 332, α6β4-Integrin, laminin-6/laminin 311, type VII collagen (5)
Linear IgA dermatosis	LAD-1, BP230 (5)
Epidermolysis bullosa acquisita	Type VII collagen (5)

## 2 Methods

### 2.1 Patients

Data of patients presenting with AIBDs at the department from 04/01/2013 to 03/31/2019 were included. The electronic patient records were screened with regard to keywords of bullous autoimmune dermatoses. 464 patients were detected, of which the diagnosis of AIBDs was finally confirmed in 242. The diagnosis or confirmation of the diagnosis of AIBDs was made on the basis of the clinical presentation and - depending on availability – serologic and immunofluorescence microscopic results. If the diagnosis was previously made externally, it had to be reconfirmed again at the dermatology clinic. For calculation of incidence, all patients with first diagnosis of a AIBDs in the given period were included. This criterion was met by 176 patients. The data analysis was done with Microsoft^®^ Excel.

### 2.2 Diagnostics

The description and localization of the patients’ skin manifestations were taken from their initial presentation at the Department of Dermatology of the University Hospital Erlangen. Only the symptoms recorded by the treating physicians were included, whereas anamnestic data given by the patients were not considered. Skin biopsies were collected (peri-)lesionally and examined both histologically and by immunofluorescence. Serological tests were performed by indirect immunofluorescence (monkey oesophagus or salt-split human skin) and ELISA (MESACUP™). When DH and celiac disease were suspected, the patient’s serum was tested for G-Transglutaminase-IgA antibodies.

### 2.3 Epidemiology

**Figure 1:**
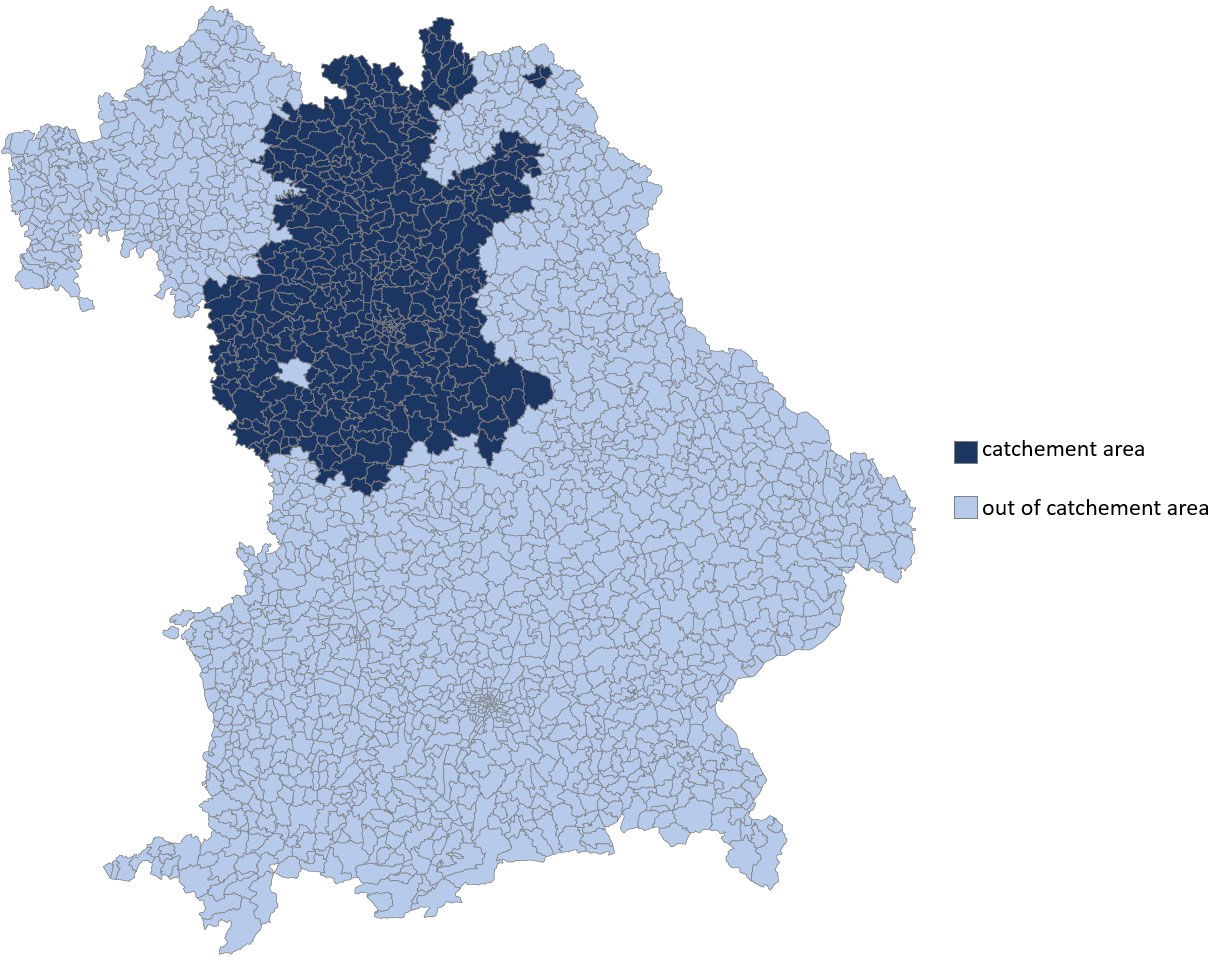
Catchment area of the Department of Dermatology at the University Hospital Erlangen within the federal state of Bavaria, determined on the basis of the Postal codes of all evaluated patients (n = 464). This covers the governorate of Middle Franconia and parts of Upper Franconia.

The catchment area of the Department of Dermatology at Erlangen University Hospital was determined on the basis of the postal codes of all patients (n = 464). Patients who came from a federal state other than Bavaria (n = 6) or from abroad (n = 2) were not taken into account. The catchment area is shown in **Figure 1**. The number of inhabitants of this defined area was 2,691,004 (50.8% women, 49.2% men). Cities from which more than two and counties from which more than four patients presented to the Department of Dermatology of Erlangen in the given period were included. The population data for the years 2013 to 2019 were provided by the Bavarian State Office for Statistics.

### 2.4 Statistics

Microsoft^®^ Excel was used for the statistical analysis of the data as well as the creation of the diagrams. Continuous variables are presented with the arithmetic mean, median and standard deviation.

## 3 Results

In total, the electronic medical records of 464 patients were analyzed. For 242 of these patients, the final diagnosis of a AIBDs was made. The following results refer to data from these 242 patients.

**Figure 2:**
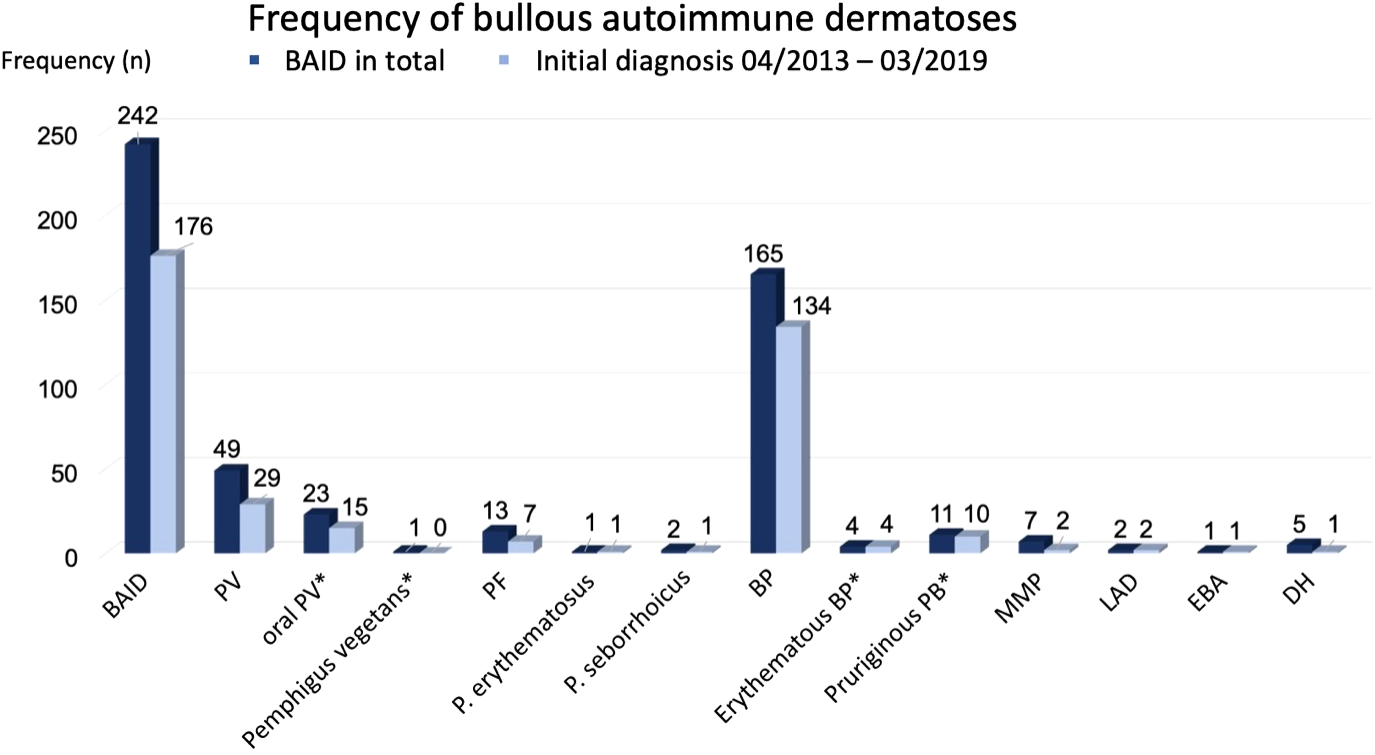
Frequency of bullous autoimmune dermatoses. Subgroups marked by * were included in main diagnose group (e.g. oral PV and PV).

In 75.2% (n = 182) the initial diagnosis was confirmed at the Department of Dermatology Erlangen. In 176 patients (72.7%), the initial diagnosis (ID) date was between 01.04.2013 and 31.03.2019. Based on this group of patients, the incidence calculation was performed. The frequency distribution of the individual AIBDs is shown in **Figure 2**. By far the most frequent diagnosis was BP (68.2%). Slightly less frequently pemphigus was diagnosed (25.6%).

The overall incidence of AIBDs was 10.9 per 1 million per year. The gender-related incidence was higher in males (11.5) than in females (10.4).

### 3.1 Pemphigus vulgaris

**Figure 3:**
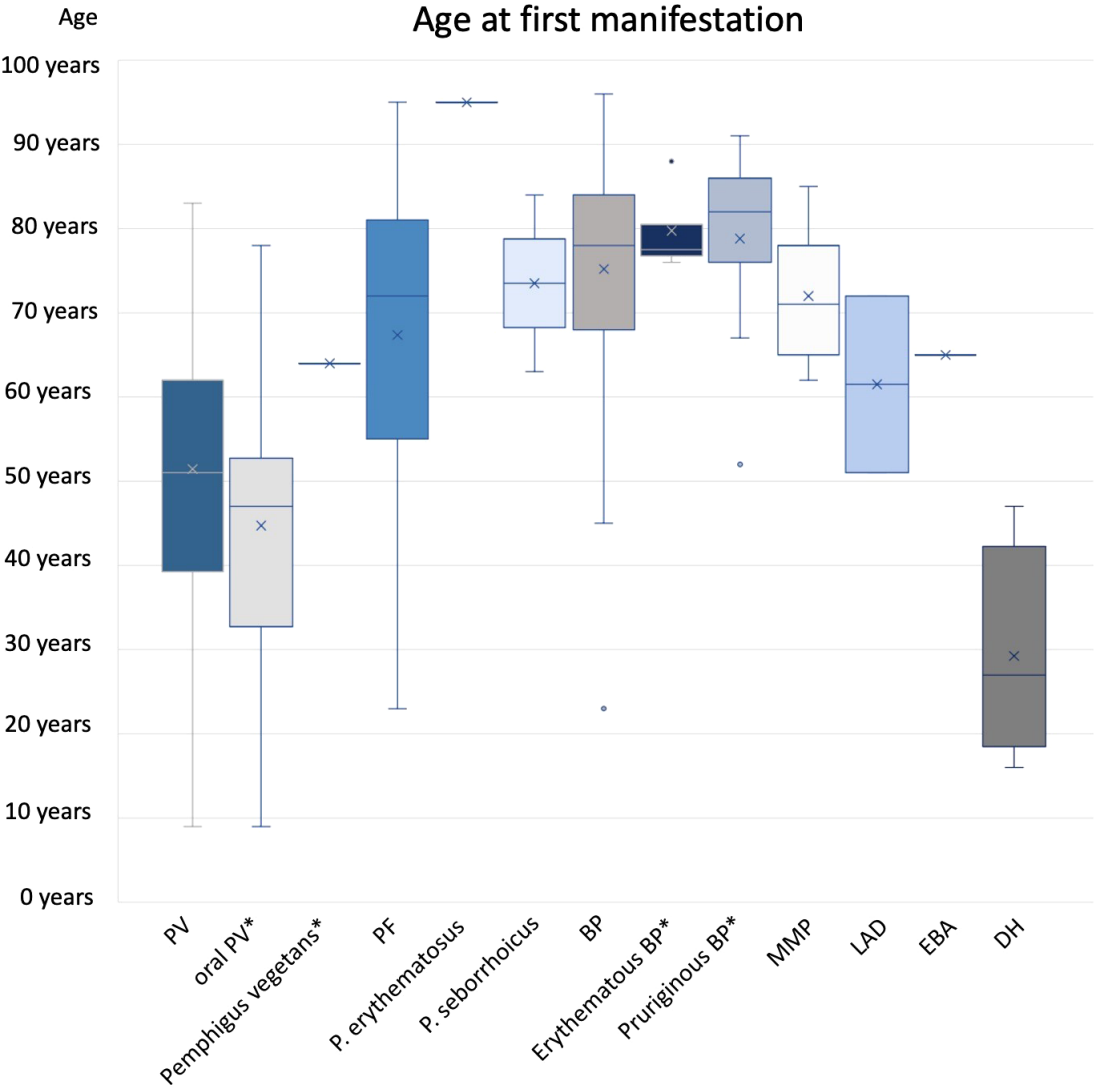
Age at first manifestation. Subgroups marked by * were included in main diagnose group (e.g. oral PV and PV).

A total of 49 cases with PV were analyzed. The incidence of PV was 1.8 per 1 million and year (**Table 2**) and showed no relation to gender (m=48,3%; f=51,7%). Patients with PV were on average 51 ± 17 years old at first manifestation (**Figure 3**). The median age was also 51 years. Altogether, a wide age range was observed between 9 to 83 years.

**Table 2:** Incidence and total number of AIBDs. The incidence is given per million and per year. Data are provided both overall and by gender (f=female, m=male).

Diagnosis	Overall Incidence	Incidence (m)	Incidence (f)	Number	Number (m)	Number (f)
Total	10,9	11,5	10,4	176	91	85
PV	1,8	1,9	1,7	29	15	14
oral PV	0,9	1	0,9	15	8	7
Pemphigus vegetans	0	0	0	0	0	0
PF	0,4	0,6	0,2	7	5	2
Pemphigus erythematosus	0,1	0	0,1	1	0	1
Pemphigus seborrhoicus	0,1	0,1	0	1	1	0
BP	8,3	8,3	8,3	134	66	68
MMP	0,1	0,1	0,1	2	1	1
LAD	0,1	0,3	0	2	2	0
EBA	0,1	0,1	0	1	1	0
DH	0,1	0,1	0	1	1	0

**Figure 4:**
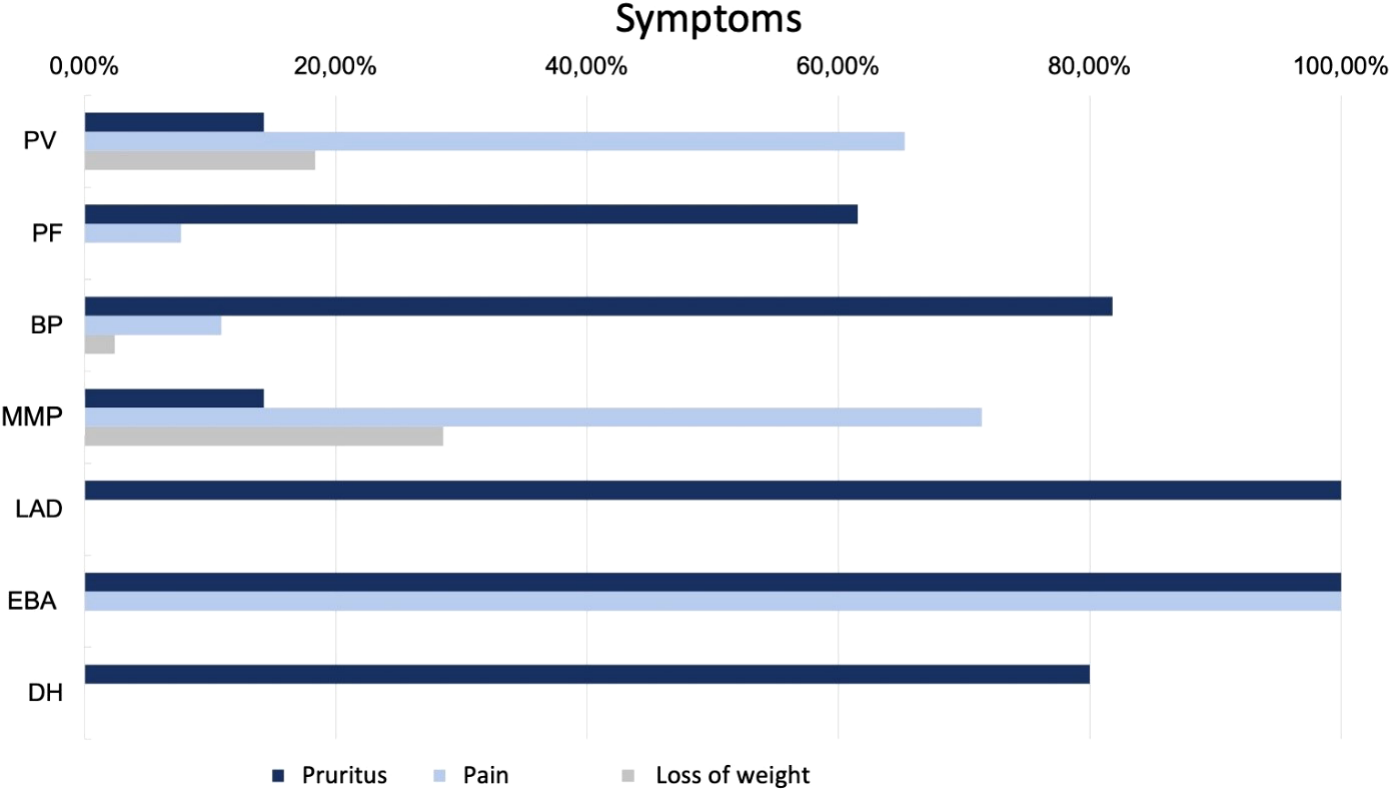
Symptoms in patients with AIBDs.

Regarding clinical symptoms, 65.3% of patients reported pain and 14.3% reported pruritus (**Figure 4**). 18.4% observed weight loss as a result of mucosal changes. 81.6% of the cases showed oral involvement, 10.2% of the anogenital mucosa and in 4.1% of the conjunctiva. In 36.7%, only mucous membranes were involved. Patients with PV disease presented most frequently with erosions (87.8%), less frequently with crusts (36.7%), plaques (24.5%) or erythema (18.4%).

Skin biopsy with subsequent histopathologic examination was performed in 30 of the 49 patients (61.2%). The histopathological findings supported the diagnosis of PV in 83.3%. 79.6% of sera were tested on monkey esophagus by IIF and 29/39 showed positive test results. DIF examination was performed in 29 of the 49 patients (59.2%). In almost three-fourths of the cases (72.4%), the result was positive with the characteristic intercellular pattern. 50.0% of serological examinations showed elevated autoantibody concentrations against Dsg 1. Concentrations of autoantibodies against Dsg 3 were elevated in 95.7% (≥20 U/ml).

48 of the 49 patients (98.0%) received systemic therapy, all of them systemic corticosteroids while 87.8% were also started on an immunomodulatory drug (azathioprine (AZA) 55.1%, mycophenolate mofetil (MMF) 22.4%, methotrexate (MTX) 8.1%, dapsone 2.0%).

### 3.2 Pemphigus foliaceus

PF was much less common, with an incidence of 0.4 per 1 million per year. The incidence in men (0.6; n=5) was three times higher than in women (0.2; n=2). At first manifestation, patients were 67± 19 years of age on average with a median of 72 years. 61.5% of patients reported pruritus in their anamnesis. Weight loss and mucosal involvement have not been observed. PF manifested as plaques and erythema in approximately three-quarters (76.9%) of patients. Scaling (61.5%), erosions (53.8%), crusts (38.5%) and blisters (15.4%) were also found. In 9 of the 13 patients (69.2%), a skin biopsy was taken for histopathological examination, which supported the diagnosis in 2/3. In 10 patients (76.9%), an IIF examination for monkey esophagus was performed, which resulted in a diagnosis in 70%. All patient specimens examined showed elevated anti-Dsg1 autoantibody concentrations. 84.6% of patients received systemic therapy (corticosteroids 76.9%; AZA 30.8%; MMF 15.4%; AZA + Dapsone 7.7%).

### 3.3 Bullous pemphigoid

BP was by far the most common diagnosis among the AIBDs studied (n=165). The incidence was 8.3 per million per year with no correlation to gender. The average age at the time of the first symptoms of the disease was 75 ± 12 years, the median age 78 years.

In BP, itching was the leading symptom in a large proportion of patients (81.8%). 10.9% suffered from pain and 2.4% reported weight loss. Oral involvement was diagnosed in 16.4% of patients. Approximately three quarters (72.1%) of the cases presented with firm blisters on the skin. Furthermore, erythema (62.4%), erosions (58.8%), plaque (38.8%), (scratch) excoriations (26.1%), and crusts (23.6%) were observed. In 152 of the 165 patients (92.1%), a tissue specimen was obtained and examined histopathologically. In 79.0%, the evaluation resulted in a diagnosis compatible with BP. IIF examination on monkey esophagus was performed in 142 of the 165 patients (86.1%). In 43.7%, a matching linear IgG deposit at the basement membrane could be found. An elevated anti-BP180 antibody concentration was detected in 79.3% of the sera tested and an elevated anti-BP230 antibody concentration in 54.8%. 73 of the patients (44.2%) showed increased autoantibody concentration against both BP180 and BP230. 74.6% received systemic corticosteroids, in 59.9% of patients combined with immunomodulatory drugs such as dapsone (48.5%) (50 -150 mg/d), azathioprine (9.6%) (0.5 - 2.0 mg/d), or MMF (1.8%) (2000 mg/d). 18 patients (11.5%) received monotherapy with dapsone and one (0.6%) received monotherapy with doxycycline.

### 3.4 Mucous membrane pemphigoid

The incidence of MMP was 0.1 per million per year. Patients presenting with MMP were on average 72 ± 8 years old. The median age was 71 years. The most common complaint was pain (71.4%), with only 14.3% reporting pruritus. In addition, nearly one-third (28.6%) of the patients reported weight loss. By definition, all patients had oral mucosal lesions. Additional involvement of the conjunctiva was documented in 57.1% and of the anogenital mucosa in 14.3%. In 85.7% a MMP manifested as erosions. Erythema (28.6%) and blisters (28.6%) were less common. Six of the seven patients (85.7%) had a biopsy taken for histopathologic examination, which supported the diagnosis in 50%. Similarly, in 50%, DIF examination could provide matching results. 71.4% were tested using IIF on monkey esophagus. With 100% positive results for IgG, this showed a higher sensitivity. In addition, four patient sera (66.7%) showed an increased concentration of autoantibodies against BP180. All 7 patients were systematically treated (85.7% corticosteroids; 42.9% AZA; 28.6% dapsone; 14.3% MMF; 14.3% cyclophosphamide).

### 3.5 Linear IgA dermatosis

LAD also had an incidence of 0.1 per million per year. The two male patients with LAD were 51 and 72 years old. Pruritus was the main symptom in both cases. One patient had oral and anogenital mucosal involvement. Both patients clinically showed mainly erythema on their skin. In one patient, the diagnosis of LAD was supported histopathologically. Evidence of linear IgA deposition in the DIF was conclusive for the diagnosis in both cases. Linear IgA deposits were also found in IIF. In addition, antibodies against BP180 were elevated. One patient was treated with internal corticosteroids while the other patient was administered dapsone.

### 3.6 Epidermolysis bullosa acquisita

During the observation period, only one 65-year-old man was diagnosed with EBA, resulting in an incidence of 0.1 per million per year. He suffered from acrally located blisters, erosions and crusts and reported itching and pain. Histopathologic evaluation of the specimen examined raised suspicion of EBA. Fluorescence microscopy showed a linear pattern along the basement membrane with IgG, IgM and C3 deposition. While IIF examination with monkey esophagus did not reveal any findings, salt split skin showed a linear IgG deposition along the blister basement. Autoantibodies against BP180, BP230, or collagen VII could not be detected in serum. The detection of collagen 7 antibodies is of high importance in the diagnosis of EBA. According to current recommendations, one of the following findings should be made when establishing the diagnosis. 1) u-serration pattern in DIF, 2) ELISA, indirect IF microscopy-based test, or immunoblotting for type VII collagen, 3) fluorescence overlay antigen mapping (FOAM) technique, or 4) direct immunogold electron microscopy (32). The investigation of the serration pattern, FOAM or direct immungold electron microscopy was not performed in this case and was not repeated due to the retrospective study design. Although there was no clear diagnostic evidence in this case, based on the available data and the clinical presentation, the diagnosis EBA seemed most likely. Therapy was initiated with systematic steroids in combination with dapsone.

### 3.7 Dermatitis herpetiformis

In summary, four patients with DH were included in this study. However, only one male patient presented with newly diagnosed disease resulting in an incidence of 0.1 per million per year. DH showed the lowest mean age at first manifestation with 29 ± 11 years and a median at 27 years. The age range was from 16 to 47 years. The main symptom was pruritus (80%). Clinically, predominant erythema (60.0%), vesicles (40.0%), and erosions (40.0%) could be objectified. A skin biopsy was taken in 4/5 patients, which confirmed the diagnosis in 75%. DIF showed IgA deposits in 75%. Serologically, G-transglutaminase IgA antibodies were detected using ELISA in 75% of the patients investigated. As first-line therapy, all five patients (100.0%) received systemic therapy with dapsone (50 - 100 mg/d).

### 3.8 Malignancy

In 7 patients (2.9%), a malignant tumor was known at the time of AIBDs diagnosis. In addition, 20 patients (8.3%) were in remission from a malignant disease. One patient (2.0%) with PV suffered from prostate carcinoma at diagnosis, and another patient was in remission of colon and prostate carcinoma. Three patients (23.1%) with PF were in remission (2x breast carcinoma, 1x prostate carcinoma). Six patients with BP (3.6%) had a known malignancy at diagnosis (2x oral squamous cell carcinoma, 1x hepatocellular carcinoma, 1x renal cell carcinoma, 1x superficial spreading melanoma; 1x basal cell carcinoma). In addition, 16 patients with BP were in remission. Here, the most common diagnoses were breast carcinoma (31.3%), prostate carcinoma (18.8%) and urothelial carcinoma (12.5%). LAD, EBA and DH patients had no malignancy in their medical history.

## 4 Discussion

This study compiled relevant epidemiological data for AIBDs in a defined area of Germany. For PV, our study showed an incidence of 1.8 per million population per year. Somewhat lower incidence values between 0.5 - 1.5 for PV disease result from studies in Lower Franconia (2001 - 2002), Lübeck (2004 - 2007) as well as France (2004 - 2013) (11, 12, 30). In the UK, much higher incidence rates of 6.8 per million per year were reported, but these were calculated from a reporting system of general practitioners and could not be examined for duplicate reporting (11, 15). The mean age of 51 ± 17 years was comparable to a study from Japan, which showed a mean age of 52.8 years at first symptoms (24). Clinical symptoms were also similar to those reported in other researches (25, 33). PF showed a significantly lower incidence at 0.4 per million per year. Here, the studies from Lübeck and Lower Franconia showed slightly higher incidences of 0.6-0.7 (11, 12). BP is the most common autoimmune bullous disorder in Central Europe. A study from France showed a regional incidence of 7.4 new cases/million/year between 1984 to 1992 (34). Our calculation showed a slightly higher incidence of 8.3 per million per year. Other regional studies in Germany showed an even higher incidence of 13.4 - 16.8 per million population per year (11, 12). BP occurs in higher age groups compared to PV. This was also shown in our study with an average of 75 ± 12 years at first symptoms. A retrospective analysis by Försti et al. on the development of the incidence of BP from 1985 to 2009 in Finland showed that it had increased 1.4-fold over the 24 years due to an aging population (35). This suggests that in the future, the incidence of these diseases will continue to increase due to higher life expectancy. The incidence of MMP in this study was significantly lower at 0.1 compared with data from France (1.25 new cases/million/year) and Lower Franconia (0.67) (10, 34). Regarding DH only one new case was diagnosed within the time limits of this investigation, leading to a low incidence of 0.1 per million and per year. The overall incidence of DH seems to vary widely depending on the region. While an incidence of 9.8 - 11 new cases per million per year has been reported in Sweden and the USA, the disease occurs only occasionally in Japan and Africa (36, 37).

LAD was also underrepresented in our study with only one case and also had an incidence of 0.1 per million per year. Studies from Lower Franconia showed a 10-fold higher incidence (11). A more frequent incidence was also reported in France with 0.48 new cases per million and year (34).

Concerning the case with EBA, we want to point out a certain diagnostic uncertainty. This again points to the need for further studies and guidelines in order to clarify this vagueness. One possibility would be to establish the examination and evaluation of the different immunodeposition patterns in DIF. Here it has already been shown that the pattern of U-serrated pattern distinguishes type VII collagen-targeted bullous diseases from other subepidermal bullous autoimmune diseases (38). A subsequent examination was not possible in our study due to the retrospective design.

In the present study, histopathological findings supported the diagnosis of PV in 83.3% and 66.6% in PF patients. This illustrates that histopathology plays an important, yet not exclusive role in the diagnostic process, but should not be used as the only method. The same is true for BP. Here, the suspicion of BP could be substantiated histopathologically by the subepidermal cleft formation in 79% of the patients (39). This needs to be confirmed by modern testing methods, such as ELISA, in the diagnosis of AIBDs.

We also demonstrated that AIBDs frequently occur in the context of malignant disease. The German and European guidelines also refers to this correlation (39–41). Therefore, it is even more important to ask for B-symptoms in patients with blistering diseases and to perform individually adapted age- and sex-specific screening examinations.

In our study, the majority of patients received internal systemic therapy with corticosteroids. Although these show rapid therapeutic success, they are accompanied by unavoidable long-term side effects. As shown in **Table 3**, different immunosuppressive or immunomodulatory therapies are established depending on the AIBD. However, these are often associated with therapy-limiting complications or show primary efficacy failure. Rituximab is approved as first-line therapy for PV. In spite of its very good efficacy, it is associated with infusion-related side effects and, most notably, with more frequent infections (42). Furthermore, the significant influence of rituximab on the response to vaccination must be taken into consideration (43). Therefore, further research into new therapeutic options is inevitable. For example, the inhibition of B-cell activation by the BAFF inhibitor lanalumab is currently being investigated (44). Targeted therapy with efgartigimod, an antagonist of the neonatal Fc receptor, was able to significantly reduce anti-Dsg antibody levels in a phase 2 clinical trial (45). CAAR-T therapy could also be a promising treatment option in the future, in particular for patients who are refractory to therapy (46). In the case of pemphigoid diseases, dapsone is often used for systemic treatment. This requires close monitoring because of the risk of anaemia or methaemoglobulinaemia, especially in elderly and multimorbid patients. Novel treatment approaches are also being investigated in BP. First reports from studies on Dupilumab, an IL4/13 antibody, which has been used for many years in the treatment of atopic eczema, show promising efficacy and safety data (47).

**Table 3:** Duration of therapy in months given as mean, standard deviation and median.

Diagnoses		PV	PF	BP	MMP	LAD	EBA	DH
Systemic Corticosteroids (0,25 - 2,5 mg/kg/d)	data available (n)	46	10	127	6	1	1	0
	mean	32.6	34.8	13.0	26.0	2.0	6.0	–
	SD	34.3	39.2	23.2	36.9	0.0	0.0	–
	median	21.0	17.0	4.0	8.0	2.0	6.0	–
Dapsone (50 - 150 mg/d)	data available (n)	11	4	109	5.	1	1	5
	mean	54.4	30.0	12.2	5.0	16.0	14.0	77.0
	SD	55.4	31.1	18.8	4.3	0.0	0.0	100.2
	median	36.0	21.5	5.0	3.0	16.0	14.0	48.0
MMF (2000 mg/d)	data available (n)	22	4	22	2	0	0	0
	mean	53.7	52.8	31.8	53.0	–	–	–
	SD	66.0	36.0	35.2	13.0	–	–	–
	median	19.0	48.5	17.5	53.0	–	–	–
MTX s.c. (10 - 15mg/w)	data available (n)	2	0	3	0	0	0	0
	mean	27.0	–	4.7	–	–	–	–
	SD	2.0	–	3.3	–	–	–	–
	median	27.0	–	4.0	–	–	–	–
MTX p.o. (5 - 10 mg/w)	data available (n)	2	0	0	0	0	0	0
	mean	39.5	–	–	–	–	–	–
	SD	30.5	–	–	–	–	–	–
	median	39.5	–	–	–	–	–	–
AZA (0,5 - 2,5 mg/kg/d)	data available (n)	28	5	33	3	1	0	0
	mean	23.6	56.0	12.6	26.3	34.0	–	–
	SD	31.9	63.8	17.2	35.8	0.0	–	–
	median	13.5	24.0	3.0	1.0	34.0	–	–
Doxycycline (200 mg/d)	data available (n)	0	0.0	7	0	0	0	0
	mean	–	–	3.4	–	–	–	–
	SD	–	–	2.9	–	–	–	–
	median	–	–	3.0	–	–	–	–
Cyclophosphamide (0,7 mg/kg/d)	data available (n)	0	0	0	1	0	0	0
	mean	–	–	–	19.0		–	–
	SD	–	–	–	0.0		–	–
	median	–	–	–	19.0		–	–
Ciclosporin (2,5 mg/kg/d)	data available (n)	2	1	0	0	0	0	0
	mean	43.5	13.0	–	–	–	–	–
	SD	19.5	0.0	–	–	–	–	–
	median	43.5	13.0	–	–	–	–	–
Doxycycline (200 mg/d) + Nicotinamid (1200 mg/d)	data available (n)	0	0	2	0	0	0	0
	mean	–	–	1.0	–	–	–	–
	SD	–	–	0.0	–	–	–	–
	median	–	–	1.0	–	–	–	–
Mycophenolic acid (1440 mg/d)	data available (n)	0	0	1	0	0	0	0
	mean	–	–	71.0	–	–	–	–
	SD	–	–	0.0	–	–	–	–
	median	–	–	71.0	–	–	–	–

The study shows some limitations, especially due to the retrospective monocentric design. The partly deviating incidences in the different studies mentioned can be explained by the overall low case numbers and different study designs. Even though the Department of Dermatology Erlangen is the only university hospital within a radius of 100 km, patients with AIBDs could have been diagnosed in other hospitals. In addition, cases may be underdiagnosed and not amply collected. To obtain more reliable data on the incidence of blistering diseases, large registries or the error-prone analysis of data from health insurance companies are needed. With regard to the diagnostics, we did not repeat the DIF examination for sample biopsies that had already been performed externally if suitable circulating antibodies were detectable by IIF or ELISA. The diagnosis was always made according to the current guidelines by a board-certified dermatologist (40, 41).

In summary, this retrospective data analysis provided epidemiologic data similar to those obtained in other regional evaluations from central Europe. AIBDs often pose diagnostic as well as therapeutic challenges to both patients and treating physicians. Further research is needed for better understand this group of disorders and identify new treatment options.

## Data availability statement

The datasets for this article are not publicly available due to concerns regarding participant/patient anonymity. Requests to access the datasets should be directed to the corresponding author.

## Author contributions

LS: Writing – original draft. VS: Writing – original draft. MS: Writing – review & editing.
